# The systemic inflammatory response, weight loss, performance status and survival in patients with inoperable non-small cell lung cancer

**DOI:** 10.1038/sj.bjc.6600466

**Published:** 2002-08-01

**Authors:** H R Scott, D C McMillan, L M Forrest, D J F Brown, C S McArdle, R Milroy

**Affiliations:** Department of Respiratory Medicine, Wishaw General Hospital, Lanarkshire ML 0DP, UK; University Department of Surgery, Royal Infirmary, Glasgow G31 2ER, UK; Department of Respiratory Medicine, Stobhill Hospital, Glasgow G21 3UW, UK

**Keywords:** non-small cell lung cancer, C-reactive protein, weight-loss, performance status, quality of life, fatigue, survival

## Abstract

The relationship between the magnitude of systemic inflammatory response and the nutritional/functional parameters in patients with inoperable non-small cell lung cancer were studied. The extent of weight loss, albumin, C-reactive protein, performance status and quality of life was measured in 106 patients with inoperable non-small cell lung cancer (stages III and IV). Survival analysis was performed using the Cox proportional hazard model. The majority of patients were male and almost 80% had elevated circulating C-reactive protein concentrations (>10 mg l^−1^). On multivariate analysis, age (*P*=0.012), tumour type (0.002), weight loss (*P*=0.056), C-reactive protein (*P*=0.047), Karnofsky performance status (*P*=0.002) and fatigue (*P*=0.046) were independent predictors of survival. The patients were grouped according to the magnitude of the C-reactive protein concentrations (⩽10, 11–100 and >100 mg l^−1^). An increase in the magnitude of the systemic inflammatory response was associated with increased weight loss (*P*=0.004), reduced albumin concentrations (*P*=0.001), reduced performance status (*P*=0.060), increased fatigue (*P*=0.011) and reduced survival (HR 1.936 95%CI 1.414–2.650, *P*<0.001). These results indicate that the majority of patients with inoperable non-small cell lung cancer have evidence of a systemic inflammatory response. Furthermore, an increase in the magnitude of the systemic inflammatory response resulted in greater weight loss, poorer performance status, more fatigue and poorer survival.

*British Journal of Cancer* (2002) **87**, 264–267. doi:10.1038/sj.bjc.6600466 www.bjcancer.com

© 2002 Cancer Research UK

## 

Lung cancer is the commonest cause of cancer death in North America and western Europe. Each year in the United Kingdom there are almost 39 000 new cases registered; only 5% are alive at 5 years (2001 CRC Cancerstats, www.cancerresearchuk.org). Most patients present with advanced inoperable disease, few are amenable to surgery and the results of radiotherapy and chemotherapy are poor.

In patients with advanced cancer, progressive weight loss is common and leads to a reduction in performance status and quality of life (O'Gorman *et al*, 1999, 2000). However, in the absence of a clear understanding of the underlying mechanisms, management of these problems has been largely ineffective (Fearon *et al*, 2001).

Therefore, it is of interest that the presence of a systemic inflammatory response (as evidenced by increased circulating concentrations of C-reactive protein) has recently been shown to be associated with loss of weight (Scott *et al*, 1996; Mcmillan *et al*, 1998) and a reduction of performance status (O'Gorman *et al*, 1998; Barber *et al*, 1999) in patients with advanced lung and gastrointestinal cancer. There is also evidence that the systemic inflammatory response is predictive of survival, independent of stage, in advanced gastrointestinal cancer (Falconer *et al*, 1995; O'Gorman *et al*, 2000).

The results of the above studies suggest that the systemic inflammatory response may contribute directly to the progressive decline in nutritional and functional status in the patient with advanced cancer. If this were so, one might expect that not only the presence, but also the magnitude, of the systemic inflammatory response would be important. However, the nature of the relationship between the magnitude of systemic inflammatory response and the loss of weight, performance status and quality of life in patients with advanced lung cancer is not clear.

The aim of the present study was to examine the relationship between the magnitude of the systemic inflammatory response and weight loss, performance status and survival in patients with inoperable NSCLC.

## MATERIALS AND METHODS

### Study design

Patients presenting between January 1995 and November 1998 with inoperable NSCLC (stages III and IV) were included in the study. All patients had cytologically or histologically confirmed disease and had no clinical or radiological evidence of infection. The patients were staged according to the American Thoracic Society TNM classification on the basis of clinical findings, chest X-ray, and where appropriate, bronchoscopy, liver ultrasound, isotope bone scan and computerised tomography of the thorax (Mountain, 1991).

The extent of weight loss, Karnofsky performance status and quality of life were measured at the time of diagnosis. A blood sample was also obtained for measurement of haemoglobin, albumin and C-reactive protein concentrations.

All patients were followed to death.

The study was approved by the Research Ethics Committee of Stobhill NHS Trust, Glasgow, UK. All subjects were informed of the purpose and procedure of the study and all gave written consent.

### Methods

#### Weight loss

Weight loss was defined as loss of more than 5% pre-illness weight in the previous 6 months.

#### Karnofsky performance status

Performance status was assessed using this numerical scale, and a score was given depending on the level of independence (Mor *et al*, 1984).

#### EORTC QLQ-C30

Different aspects of quality of life were assessed using this cancer specific 30-item questionnaire, which has six functional scales (physical, role, emotional, cognitive, social, global health status) and several questions relating to a range of physical symptoms (Aaronson *et al*, 1993). Patients marked to what extent each statement applied to them.

#### Blood parameters

Routine laboratory measurement of haemoglobin, albumin and C-reactive protein concentration was carried out. The coefficient of variation for these methods, over the range of measurement, was less than 5% as established by routine quality control procedures.

### Statistics

Data are presented as median and range. Where appropriate, comparison of patient groups was carried out using the Kruskal-Wallis test for analysis of variance.

Survival analysis was performed using the Cox proportional hazard model with patients' age, sex, stage, histological type, weight loss, haemoglobin, albumin, C-reactive protein, Karnofsky performance status and EORTC QLQ-C30 subscales as prognostic variables.

Multivariate survival analysis was performed using a stepwise backward procedure to derive a final model of the variables that had a significant independent relationship with survival. To remove a variable from the model, the corresponding *P*-value had to be greater than 0.10. Survival curves were analysed by the Kaplan-Meier method. In terms of the relationship between the systemic inflammatory response and survival it was assumed that any palliative treatment following measurements at diagnosis had no significant impact on the duration of survival.

Analysis was performed using SPSS software (SPSS Inc., Chicago, Illinois, USA).

## RESULTS

The characteristics of patients with inoperable NSCLC (*n*=106) are shown in Table 1

**Table 1:**
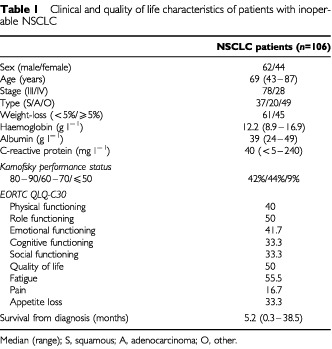
Clinical and quality of life characteristics of patients with inoperable NSCLC

. The majority of patients were male, over the age of 60 years and had stage III disease. Approximately 40% had more than 5% weight loss and almost 80% had elevated circulating C-reactive protein concentrations (>10 mg l^−1^). The median Karnofsky performance status was 70. All scores of the EORTC-QLQ C30 functional scales were poor (50 or less); also, the fatigue scale scores were poor (50 or more). Median survival from the time of diagnosis was 5.2 (range 0.3–38.5) months.

On univariate analysis, stage (*P*=0.020), tumour type (*P*<0.001), albumin (*P*=0.002) and C-reactive protein (*P*=0.004) concentrations, Karnofsky performance status (*P*<0.001), physical functioning (*P*=0.013), fatigue (*P*<0.001), pain (*P*=0.045) and appetite loss (*P*=0.004) were significant predictors of survival. On multivariate analysis, only age (HR 1.040 95% CI 1.009–1.072, *P*=0.012), tumour type (HR 1.655, 95% CI 1.200–2.282, *P*=0.002), weight loss (HR 0.605 95% CI 0.362–1.012, *P*=0.056), C-reactive protein (HR 1.783 95% CI 1.009–3.151, *P*=0.047), Karnofsky performance status (HR 0.964 95% CI 0.942–0.987, *P*=0.002) and fatigue (HR 1.011 95% CI 1.000–1.022, *P*=0.046) remained significant independent predictors of survival.

Patients were then grouped according to the magnitude of the C-reactive protein concentrations namely ⩽10, 11–100 and >100 mg l^−1^ as previously described (Mcmillan *et al*, 2001; Table 2

**Table 2:**
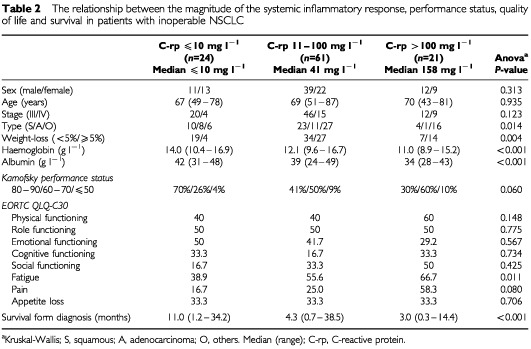
The relationship between the magnitude of the systemic inflammatory response, performance status, quality of life and survival in patients with inoperable NSCLC

). The distribution of sex, age and stage was similar between these groups. An increase in the magnitude of the systemic inflammatory response was associated with an increase in the relative proportion of non-adenocarcinoma (*P*=0.014), increased weight loss (*P*=0.004), reduced haemoglobin (*P*<0.001) and albumin concentrations (*P*<0.001) and reduced performance status (*P*=0.060). With the exception of fatigue (*P*=0.011), there was no significant relationship between the magnitude of the systemic inflammatory response and the EORTC QLQ C-30 subscale scores.

Median survival ranged from 11 months in those with a C-reactive protein concentration of 10 mg l^−1^ or less, to 3 months for those with a C-reactive protein concentration greater than 100 mg l^−1^ (HR 1.936 95% CI 1.414–2.650, *P*<0.001).

## DISCUSSION

In the present study of patients with inoperable NSCLC, the presence of a systemic inflammatory response was associated with increased weight loss, a reduction in performance status, increased fatigue and reduced survival. This is consistent with previous studies, in patients with lung cancer, showing that the presence of a systemic inflammatory response is associated with an increase in resting energy expenditure (Staal-van den Brekel *et al*, 1995; Scott *et al*, 2001), loss of lean tissue (Mcmillan *et al*, 1998; Simons *et al*, 1999), decreased performance status and survival (Martin *et al*, 1999).

If the relationship between the presence of a systemic inflammatory response was causal, an increase in the magnitude of the systemic inflammatory response should impact on weight loss, performance status and survival. It is therefore of interest that in the present study, exponential increases in circulating concentrations of C-reactive protein were associated with increased weight loss, reduced performance status, increased fatigue and decreased survival. These results would suggest that a common mechanism underlies the progressive nutritional and functional decline of patients with inoperable NSCLC, which ultimately impacts on survival.

The mechanism by which a systemic inflammatory response might influence cancer survival is not clear. However, it is known that as part of the systemic inflammatory response, there is a release of pro-inflammatory cytokines and growth factors (Fearon *et al*, 1991; Moldawer and Copeland, 1997) some of which produce metabolic disturbances (Mcmillan *et al*, 1994; Barber *et al*, 2000) and loss of lean tissue (Mcmillan *et al*, 1998). The presence of these factors may also promote tumour growth (Moldawer and Copeland, 1997; Abramovitch *et al*, 1999) which, in turn, may further stimulate the systemic inflammatory response.

The specific factors which determine the magnitude of the systemic inflammatory response evoked in patients with inoperable NSCLC are not clear. The systemic inflammatory response may be secondary to tumour necrosis or local tissue damage caused by the tumour-host cell interaction. It is of interest that, although in the present study the magnitude of the systemic inflammatory response was not associated with disease stage it was associated with a decrease in the proportion of adenocarcinomas. It may be that the non-adenocarcinoma phenotype preferentially stimulates a systemic inflammatory response.

In summary, the results of the present study indicate that the majority of patients with inoperable NSCLC have evidence of a systemic inflammatory response at the time of diagnosis. Furthermore, the greater the magnitude of the systemic inflammatory response, the greater the weight loss, the poorer the performance status, the greater the fatigue and the poorer the survival.
